# High non-compliance rate among presumptive tuberculosis cases referred from peripheral health facilities in silti district of Southern Ethiopia: a mixed methods study

**DOI:** 10.1186/s13690-023-01071-w

**Published:** 2023-04-04

**Authors:** Habtamu Milkias, Delenasaw Yewhalaw, Gemeda Abebe

**Affiliations:** 1 School of Medical Laboratory Sciences, Institute of Health, Jimma University, Jimma, Ethiopia; 2 Mycobacteriology Research Centre, Jimma University, Jimma, Ethiopia; 3 Tropical and Infectious Diseases Research Center, Jimma, Ethiopia; 4 Federal Ministry of Health, Addis Ababa, Ethiopia

**Keywords:** Compliance, Presumptive TB cases, Tuberculosis, Referral, Ethiopia

## Abstract

**Background:**

For presumptive Tuberculosis (TB) case referral to be effective, most of the referred cases need to present themselves to health facilities for assessment and testing. Otherwise, cases of TB could be missed, and these cases are at an increased risk of delayed diagnosis, complications and death. Further, their care incurs significantly higher costs. This study assessed referral compliance as well as factors attributable to compliance/non-compliance to referral of presumptive TB cases in Silti district, Southern Ethiopia.

**Method:**

We applied a mixed design involving both quantitative and qualitative methods. A randomly selected sample of 384 presumptive TB cases referred between January, 2014 and July 2021 were included in this study from the records of 12 health posts. Purposefully selected presumptive TB cases and Health Extension Workers were also interviewed to get in-depth information on the reasons for compliance and non-compliance to referral. STATA version 14 was employed to model the data using logistic regression. Qualitative data were analyzed using thematic content analysis.

**Results:**

Of the 384 referred presumptive TB cases, close to 49% did not present themselves to the referral facilities. About 66% (n = 249) of the referred cases were women, and 62% (n = 119) of those who complied to referral were women. In multivariate analysis, cough [AOR = 3.4, 95%CI: 1.54–7.32], and chest pain [AOR = 2.7, 95%CI: 1.45–5.05] were independent predictors of compliance to referral. Nearly 5.5% (n = 21) of TB cases of all types were identified. The qualitative data analysis revealed that severe disease symptoms, HEW’s recommendations, and social issues as reasons improving compliance while personal and social factors, financial problems, lack of awareness about TB and transportation were reasons impeding compliance to referral.

**Conclusion:**

Our study showed a high level of non-compliance to referral among referred presumptive TB cases. We also found that more women were referred and also complied with the referral. Strengthening community awareness about the disease symptoms and the existence of free treatment, addressing misconceptions about TB, supporting the elderly and disabled, and checkup house visits after referral could improve compliance to referral.

**Supplementary Information:**

The online version contains supplementary material available at 10.1186/s13690-023-01071-w.

## Background

Nearly 10 million new TB cases were identified in 2020 with an estimated 4.1 million undiagnosed cases in the same year according to a report by the World Health Organization (WHO) [1]. Undiagnosed TB cases are likely to seek care at a delayed time thereby increasing their chances of undesirable health outcomes and higher cost of care [2]. Furthermore, such cases enable continued propagation of the disease in the community [2, 3]. National TB control programs are hence, mandated with early identification of TB cases and the prompt initiation of chemotherapy [4, 5]. Relying on identification of cases through patients or clients appearing to healthcare facilities by themselves seeking care either for suggestive symptoms of TB or another service, is not effective as described in various studies [6–8]. Cognizant of this, the WHO included systematic screening of TB among several high-risk groups in its End TB Strategy [9] which was followed by an issuance of a guideline for prioritization of the risk groups and the screening approaches to follow [3]. Studies consistently indicate that active and intensified case finding strategies yield more TB cases compared to passive case finding [10–12].

Despite recommendations for active case finding through systematic screening of high-risk groups, facilities in low and middle income countries struggle with implementation of the strategy [6, 13, 14]. Various factors are attributed including inadequate training, scarcity of workforce, illiteracy and inadequate community awareness about TB. Unavailability or insufficiency of diagnostic facilities [15] and resources [14] are the other key factors limiting its implementation. Ethiopia included recommendations for systematic screening of TB among predetermined and prioritized groups in its national guideline in 2017 [16]. However, studies indicate that similar challenges observed in other countries exist in Ethiopia too. For instance, about 30% of all health centers do not have a TB screening service [17]. TB case management capacity is also poor among a high proportion of the health centers in the country. According to a national survey, below 10% of staff in health centers reported adequate knowledge and skill of diagnosing and managing TB [17, 18]. Scarcity of trained staff and poor TB screening practices contribute to a high rate of missed TB cases in healthcare facilities [18, 19].

In Ethiopia, TB prevention and control activities at the community level are integrated with the Health Extension Program (HEP). HEP is a primary health care service introduced by the Ministry of Health in 2003 with the aim of rapidly expanding health care services to the inaccessible and underserved rural communities. The program was originally launched in the country’s four big agrarian regions (Amhara, Oromia, SNNPR and Tigray). Later, it was expanded to the pastoralist communities in 2006 and urban areas in 2010. Health posts at the lowest level of the Ethiopian healthcare tier system are the facilities where HEP services are delivered in addition to services provided through regular house hold visits. Services are provided by at least two female, college trained and salaried Health Extension Workers (HEWs) and is based on nationally developed packages tailored to the varying situations and requirements of the different regions in the country [20]. TB prevention and control is one of the 16 packages of the program. TB related services by HEWs include education and creating awareness in the community on TB, identification and referral of presumptive TB cases and treatment adherence and follow up [16, 21, 22]. Additionally, they are involved in identification and referral of MDR-TB suspects, tracing of defaulters and identifying and facilitating treatment adherence supporters [22].

Only 12 countries including Ethiopia account to 75% of the 4 million missed TB cases annually in the world. Studies indicate more than a third of TB cases are not diagnosed and notified each year in Ethiopia [23, 24], which poses a great challenge for the national TB program. Case detection rate was at 71% in 2020 according to the data by the World Bank. Hence, there is an urgent need to improve referral and case notification rate to achieve the End TB target.

Presumptive case referral by HEWs could help improve case detection rate but that’s possible provided that majority of the referred cases comply to the referral and appear to the referred facilities for further screening and testing. Previous studies have mostly focused on improving active case finding and access to TB services but there hasn’t been prior study on compliance to referral which is a key part to improve case finding and treatment initiation. Hence, this study investigated the rate of non-compliance to referral by HEWs as well as the reasons behind compliance and/or non-compliance to referral in the Silti district of Southern Ethiopia.

## Methods

### Study setting and period

The study was conducted in 12 kebeles (the lowest administrative unit) of Silti district in Southern Nations, Nationalities and Peoples Region (SNNPR) in Ethiopia from February to July, 2021. The district’s capital is called Kibet and is located 147 KMs South of the capital Addis Ababa, the Ethiopian capital. The projected total population of the district for the year 2021 was 201,195 of whom 99,510 were men and 101,685 were women. Most of the kebeles in the district are rural except six which are within the capital of the district. Most of the rural population in the district dwell either in small thatched roof circular huts or houses with corrugated iron sheet covers. The district has one hospital, four health centers and 31 health posts. The total health workforce in the district including HEWs is 305. Health posts in the kebeles provide community level TB prevention and control services such as health education, DOTS (Directly Observed Treatment Short Course) supervision, presumptive TB case identification and referral.

### Study design

The study employed a mixed approach involving both quantitative and qualitative methods. A cross sectional design was used to obtain quantitative data regarding the demographic, clinical characteristics, compliance to referral and TB status of the presumptive TB cases from referral registers in the health posts, log books and unit TB registers found in the referral hospital. In depth interviews were made with purposefully selected presumptive TB cases and HEWs to examine the reasons for compliance and non-compliance to referral by HEWs.

### Sample size and sampling method

We used presumptive TB cases referred by HEWs from 12 health posts of Silti district (which refer to Kibet Primary Hospital) as the source population and a representative sample of the referred presumptive TB cases were identified to be included as a study population. Since the population of interest is single (referred presumptive TB cases), a single population proportion formula was used to calculate a sample size of 384 with the assumption that 50% of the referred presumptive TB cases have complied (as there was not prior estimate) and appeared to the referral facility for further assessment and diagnostic testing with 95% level of confidence and 5% precision.

A simple random sampling technique was used for the quantitative study to select the study participants. Initially, list of all referred presumptive TB cases between Jan, 2014 and July, 2021 were identified from the referral registers of each health post and were given unique identification numbers. Using this list, the number of referred cases to be included in the study was allocated proportionally for each health post based on their total number of referrals. The study participants were then randomly selected using a random number function on Microsoft Excel. For the qualitative study, 14 presumptive TB cases were purposefully selected considering saturation (seven from those who complied to referral and seven from those who didn’t comply to the referral). Those aged 18 or older who could give details of their reasons were included into the qualitative study. Additionally, two HEWs were included for the qualitative study to get their version of reasons for compliance and/or non-compliance to referral among presumptive TB cases.

### Data collection and quality assurance

For the quantitative data collection, a data abstraction tool was developed and pretested in a health post and a hospital out of the study area to ensure reliability and avoid confusion among data collectors. The tool was first developed in English and then translated into Amharic, which is the working language in the study area. The tool was developed with the intention to collect data from registers on the socio-demographic characteristics, clinical signs and symptoms during initial screening, compliance to referral, TB status and treatment outcome of the participants. Compliance to referral is verified by corroborating the identification and socio-demographic data from the referral registers with data captured on a log book in the referral hospital. Referred presumptive TB cases whose data are not captured in the hospital log book are considered non compliers (i.e., didn’t appear to the referral facility for TB assessment and testing) as all referrals from catchment health posts by HEWs are registered in the log book. For the qualitative data collection, a semi-structured in-depth interview questionnaire was used to further understand the detailed reasons behind compliance or non-compliance to referral by HEWs. The data collectors were healthcare professionals having at least a BSc and an experience of working in TB clinic either in health centers or hospitals. They were given an orientation on the data extraction tool for quality assurance and consistency.

### Study variables

The main outcome of interest in the current study is compliance to referral by HEWs of presumptive TB cases i.e., appearance to the referral facility for further TB assessment and diagnostic testing. A presumptive TB case is defined as an individual who presents with persistent cough of two weeks or more or at least two of the following symptoms: fever of more than 2 weeks, drenching night sweats or unexplained with loss of 1.5 kg in a month [16]. The other outcome of interest is the yield of TB which is defined as the proportion of TB among presumptive TB cases who have been assessed and tested for TB. The independent variables considered for analysis were age of the participant, sex, symptoms during screening (including cough, bloody sputum, chest pain, low grade fever, night sweating, loss of appetite and weight loss), presence of TB patient in the family, kebele/health post of referral, year of referral, TB status, type of TB, treatment category, body mass index, year of treatment, HIV status, presumptive MDR TB status, rifampicin resistance status and treatment outcome.

### Data analysis

The quantitative data was first entered into Microsoft Excel 2019 and was then exported into STATA 14 (Stata-Corp, College Station, Texas, USA) for analysis. Descriptive statistics such as mean, standard deviations, frequency and proportions were calculated for the outcome and predictor variables. Chi square and bivariate logistic regression analyses were made to investigate the association between the outcomes and independent variables. Predictor variables yielding p-value < = 0.25 were selected for inclusion into the multivariable logistic regression model. A p-value of less than 0.05 was considered statistically significant in the multivariable logistic regression. Model fitness was checked using Hosmer Lemeshow Goodness of fit test, classification table and the ROC curve. We have also checked collinearity of the included variables using variance inflation factor and tolerance. For qualitative data analysis, audio records of the in-depth interviews were first transcribed into Amharic (one of the local languages) and then translated into English. Finally, Matrix analysis was conducted using Microsoft excel to identify themes from the qualitative data.

## Results

### Demographic characteristics and clinical symptoms at the time of referral

A total of 384 presumptive TB cases referred by HEWs were included from 12 health posts. They had a mean age of 38 years with a standard deviation of 19 years. Among the referred presumptive TB cases, 7.6% (n = 28) were children less than 5 years. About 66% (n = 249) of the referred presumptive TB cases were female. The female to male ratio was 1.9: 1 (Table 1).

**Table 1:** Demographic data and clinical symptoms of referred presumptive TB cases in Silti District, Southern Ethiopia, 2014–2021

Variables (n = 384)		Frequency (%)	Variables (n = 384)		Frequency (%)
Sex	Male	130 (34.3)	Weight loss	No	162 (42.7)
	Female	249 (65.7)		Yes	217 (57.3)
Age	Five year and less	28 (7.5)	Known TB case in the family	No	204 (57.6)
	6–20 years	56(15.1)		Yes	150 (42.4)
	21–40 years	112(30.2)	Year of referral	2014	17 (4.4)
	41 and above	175(47.2)		2015	2 (0.5)
Cough	No	53 (13.8)		2016	1 (0.3)
	Yes	330 (86.2)		2017	60 (15.6)
Blood in the sputum	No	278 (73.4)		2018	127 (33.1)
	Yes	101 (26.6)		2019	55 (14.3)
Chest pain	No	112 (29.5)		2020	77 (20.1)
	Yes	268 (70.5)		2021	45 (11.7)
Low grade fever	No	91 (24.1)			
	Yes	287 (75.9)			
Night sweating	No	116 (30.5)			
	Yes	264 (69.5)			
Loss of appetite	No	85 (22.4)			
	Yes	295 (77.6)			

About 42.4% (n = 150) presumptive TB cases indicated that there was at least one known TB case in their family prior to their referral. The trend of referral in the early years of implementation i.e., from 2014 to 2016 shows a fewer proportion of referrals; 4.4%, 0.5%, and 0.3% respectively. But since 2017, the proportion of referrals has increased until it slightly fell back again in 2021 to just 11.7% (Table 1).

### Compliance of presumptive TB cases to referral by HEWs

Nearly 51% (n = 196) of the referred presumptive TB cases by HEWs have complied and presented themselves to the referral facility. More women (61.7%) complied to referral by HEWs compared to men. But, among those who didn’t appear to the referral facility, the majority (70%) remained to be women. Of the presumptive TB cases who complied, 5.8% (n = 11) were children aged one year or less. About 35.8% (n = 69) of cases who reported blood-stained sputum complied to referral. Only 48% (n = 78) have appeared to the referral facility among the referred cases from households having a known TB case. In 2017, the proportion of compliance to referral was 43.3%. This proportion increased to 67% in 2018 and 89% in 2019. But in 2020 and 2021, the proportion of compliance decreased to 25.5% and 35.5% respectively.

### Factors associated with compliance to referral

In bivariate logistic regression analysis, the odds of compliance to referral by presumptive TB cases was significantly associated with having symptoms of cough and chest pain. Year of referral also had a significant association with compliance to referral on crude analysis with higher odds of compliance in the years 2018 and 2019 compared to the year 2017. In contrast, the odds of compliance were lower in the year 2020.

Controlling for chest pain and year of referral, presumptive TB cases with a cough had about three times higher odds of appearing and getting tested in the referral facility compared to those who didn’t report the symptom (AOR = 3.4, 95%CI: 1.54–7.32). Likewise, presumptive TB cases who reported chest pain had nearly three times higher chances of compliance to referral as compared to the cases without the symptom, after adjusting for cough and year of referral (AOR = 2.7, 95%CI: 1.45–5.05). The likelihood of compliance in 2018 was three times higher compared to the year 2017 (AOR = 3.3, 95%CI; 1.66–6.47). Similarly, presumptive TB cases compliance was about 19 times higher in 2019 compared to 2017 (AOR = 19.1, 95%CI: 6.28–57.93). However, the probability of compliance was 53% lower in the year 2020 compared to 2017 (AOR = 0.47, 95%CI: 0.22–0.99). Factors such as weight loss, loss of appetite, fever, blood-mixed sputum, night sweating and presence of a known TB case in a household had no significant association with compliance to referral (Table 2).

**Table 2:** Factors associated with compliance to referral by presumptive TB cases in Silti District, Southern Ethiopia, 2014–2021

Variable (n = 384)		Complied to referral		COR (95% CI)	AOR (95%CI)
		No (%)	Yes (%)		
Sex	Male	56 (30.1)	74 (38.3)	1	1
	Female	130 (70.0)	119 (61.7)	0.7 (0.45–1.06)	1.01 (0.53–1.91)
Age category	<=5 year	17 (60.7)	11 (39.3)	1	1
	6–20 years	22 (39.3)	34 (60.7)	2.4 (0.94–6.05)	3.2 (0.90-11.59)
	21–40 years	51 (45.5)	61 (54.5)	1.8 (0.79–4.30)	2.2 (0.66–7.07)
	41 and above	90 (51.4)	85 (48.6)	1.5 (0.65–3.29)	1.8 (0.56–5.62)
Cough	No	13 (6.9)	15 (7.7)	1	1
	Yes	175 (93.1)	180 (92.3)	1.9 (1.03–3.38)	3.4 (1.54–7.32)**
Bloody sputum	No	124 (66.7)	124 (64.2)	1	1
	Yes	62 (33.3)	69 (35.8)	1.2 (0.77–1.92)	1.07 (0.53–2.14)
Chest pain	No	42 (22.5)	36 (18.7)	1	1
	Yes	145 (77.5)	157 (81.4)	3 (1.88–4.75	2.7 (1.45–5.05)**
Low grade fever	No	40 (21.5)	32 (16.7)	1	1
	Yes	146 (78.5)	160 (83.3)	0.9 (0.53–1.36)	0.6 (0.27–1.24)
Night sweating	No	38 (20.4)	34 (17.5)	1	1
	Yes	148 (79.6)	160 (82.5)	1.2 (0.79–1.91)	1.2 (0.61–2.36)
Loss of appetite	No	27 (14.4)	46 (24.2)	1	1
	Yes	160 (85.6)	144 (75.8)	0.9 (0.57–1.54)	0.7 (0.29–1.45)
Weight loss	No	36 (19.3)	42 (22.2)	1	1
	Yes	151 (80.7)	147 (77.8)	1 (0.67–1.51)	0.9 (0.51–1.66)
Known TB case in the family	No	100 (54.4)	90 (53.9)	1	1
	Yes	84 (45.6)	77 (46.1)	0.9 (0.63–1.46)	0.8 (0.47–1.55)
Year of referral	2017	34 (18.2)	26 (13.4)	1	1
	2018	50 (26.7)	77 (39.7)	2.7 (1.79–7.48)	3.3 (1.66–6.47)**
	2019	4 (2.1)	50 (25.7)	10.7 (3.96–28.73)	19.1 (6.28–57.93)**
	2020	54 (28.9)	23 (11.8)	0.43 (0.22–0.88)	0.47 (0.22–0.99)**

** significant at p < 0.05

### The yield of TB and treatment outcome

Among the referred presumptive TB cases, 5% (n = 21) TB cases were identified. The figure became 10.8% among those who appeared to the referral facility and got tested for TB. In terms of the types of TB, 71.4% (n = 15) were smear positive, while 14.3% (n = 3) were smear-negative and 14.3% (n = 3) were extrapulmonary tuberculosis (EPTB) cases. Of the identified TB cases 85.7% (n = 18) were classified as new cases where as 14.3% (n = 3) were categorized as failures. In terms of the treatment year, 14.3% (n = 3) were treated in 2018, 66.7% (n = 14) were treated in 2019 and 19.1% (n = 4) were treated in 2020. The patients had an average Body Mass Index (BMI) of 18.5 (SD = 3). All of the TB cases were non-reactive up on Human Immunodeficiency Virus (HIV) testing. About 18.8% (n = 3) cases were considered presumptive Multidrug Resistant TB (MDR TB) cases but no rifampicin resistance was found up on testing with GeneXpert. In terms of treatment outcome, 37.5% (n = 6) of cases completed treatment, 43.8% (n = 7) got cured, 12.5% (n = 2) died, 6.3% (n = 1) had a failure of treatment while 23.8% (n = 5) cases had no recorded treatment outcome.

### Reasons for compliance of presumptive TB cases to referral by HEWs: perceptions and practices

A total of 14 previously referred presumptive TB cases were interviewed in-depth to reach sampling saturation. The respondents’ mean age was 35 years with a standard deviation of 9 years. Most of the respondents either had no or primary education. In terms of employment 70% were unemployed while the rest practiced farming. All of the respondents were Muslim. The distance from the respondent’s residence to the referral facility ranged from 1 Km to 14 Km. The average number of months since referral is 6 months. Additionally, two HEWs were interviewed to understand their view. Analysis of the qualitative data has categorized the reasons for appearing to a referral facility for further assessment and testing after referral by HEWs (i.e., compliance to referral) into three main themes. These are severe disease symptoms, conforming to HEWs advice and facing social issues in the family or community.

The most common reason described by respondents for compliance was severity of the disease symptoms. Some of these symptoms that alerted them to seek immediate attention include, experiencing severe persistent cough, seeing blood mixed in the sputum, progressive weight loss, difficult breathing and inability to eat or drink. One participant described the urge she felt to seek care as follows: “*I used to be healthy and had a normal weight before I started to see these symptoms. I had severe cough, chest pain, loss of appetite and inability to sleep for a long time. I have been treated repeatedly for about a year but my weight continued to decrease until I looked so small like a child. I never used to look like this. Then, the HEW referred me to the hospital to get tested for TB after which I was told that I have the disease and I started treatment.”* Some respondents also indicated the reason for compliance as simply seeking for health service out of concern for one’s own health.

The other frequently mentioned reason for compliance was adhering to HEWs advice for immediate testing and treatment. In two cases however, this reason was cited in conjunction with the inability to see improvement in their condition/symptoms despite taking some actions. One respondent said *“I was trying to cure my health problem with both drugs prescribed from clinics and traditional remedies such as taking garlic, egg yolk, honey and hot drinks but my condition kept worsening. But at some point, the HEW advised me to get tested for TB and that is when I decided to obey to her advice.”*

The third major theme discovered is social issues as a reason for complying to the referral. For instance, one respondent described her experience in the community that forced her to visit a healthcare facility as follows: *“…the villagers in my community heard that I had symptoms of the disease. One day, they warned me that I must visit a healthcare facility together will all my family members or else I could infect the other community members implying that they intend to avoid any social interaction with me and my family members. That’s when I felt the pressure to go and get tested with all my family.”* In another instance, a respondent described being unable to have a child in his marriage forced him to visit a healthcare facility after being referred by a HEW. He said “*After repeatedly failing to appear to a healthcare facility after referral by HEWs, I started to have swelling and pain on my testicles and difficulties during urination. At the same time, my wife wasn’t able to conceive for more than a year. This made me to visit a hospital and upon assessment, I was told to have an extrapulmonary TB affecting my testicles and that it may have caused me infertile.”*

### Reasons for non-compliance of presumptive TB cases to referral by HEWs

Concerning the reasons for non-compliance to referral, the result has revealed four main themes. These are personal and social issues, financial problems, lack of awareness about TB and transportation issues. Personal and social issues further have sub-themes which includes prioritizing other competing daily activities at home, having a physical disability and being non-ambulatory, fear of community stigma, addiction/substance use and having an attitude of low self-worth. Transportation issue has two sub themes namely lack of transport and the need to travel long distance to the referral facility.

By far the most frequently cited reasons for not complying to referral fall under the personal and social issues category. Prioritizing other competing daily activities at home is cited frequently as a reason for not complying to referral. Some respondents described presence of minors and dependents at home who need care and support of others in the family there by taking precedence over attending a healthcare facility. Others also indicated farming activities especially during rainy seasons. Individuals with physical disability found to be presumptive TB cases also find it difficult to attend a healthcare facility after referral if they are not supported as are old people who are weak and unable to walk according to the respondents. Community stigma is another factor described by the respondents as a reason hindering compliance to referral. One HEW said “…*there is a special saying in our community when a person is found to have TB.* They say *‘this person has contracted TB’ and this is scary for other people in the neighborhood. It implies that it’s dangerous to approach the person regardless of treatment initiation*.” Addiction and substance use such as chewing Khat (*Katha edulis*), shisha and others have also been described as a reason for non-compliance to referral. Personal negligence and having low self-worth were other reasons identified and have been described by some respondents together with addiction. For instance, one respondent said “*… I had a negligent attitude about myself and my health and also, I had addiction to khat and cigarette. Because of this, I failed to get tested at the hospital although I was referred repeatedly by the HEWs for about a year. This resulted in my disease being disseminated to other parts of my body.”*

Financial problem was second as frequent reasons reported for non-compliance to referral. The respondents said phrases like “personal problems, problems at home, lack of money, inability to borrow money for travel and other costs and fear of higher out of pocket expenses” to indicate financial problems as reasons for not promptly complying to referral by HEWs. Another aspect of financial problems is the community’s perception about eating habits during TB medication. “*It is believed that patients on TB medication are required to consume a lot of food and hence strains the person/family financially…*” said another HEW.

Lack of awareness about TB and the associated misconceptions were the third theme revealed by the study. Unawareness about the consequences of the disease without treatment, inadequate understanding about seriousness of the disease and availability of treatment were the common descriptions used by the respondents to explain awareness issues about TB that led them to not comply to referral straightaway. A few participants told that the HEWs didn’t explain in detail about the disease including the fact that the laboratory testing and treatment are free of charge. Transportation issues were also repeatedly indicated as reasons by the participants for not complying to referral. While absence of transportation was a problem for some of the respondents, others informed the need to travel long distances to the referral facility. The later reason was cited by a few respondents even when there are available means of transportation. Another reason closely related to this was the hesitance of the individuals to make a follow up visit to the facility after initial negative result as mentioned by one HEW. She described that they don’t want repeated travel to the facility.

### Information provided by HEWs at the time of referral

While some participants described that adequate information were given during the time of referral by HEWs, some indicated that they didn’t get sufficient information regarding the disease, the treatment and the need for prompt testing at a referral facility. Some of the information provided during the time of referral explained by the participants include the threat of the disease if not treated, the need to cover nose and mouth and isolating sleeping place at home, use of private utensils for eating and drinking until initiation of treatment. Apart from these, the participants described that the HEWs had explained them about free laboratory testing and treatment of TB, the need to take the medication every day without discontinuation as described by the healthcare workers and also the necessity of eating a balanced diet during the treatment period.

## Discussion

This is the first study that has evaluated the effectiveness of community based presumptive TB case referral in Ethiopia. In this study, we showed that a high proportion of referred presumptive TB cases (49%) did not present themselves to the referral facility. We also found that more women were referred (65.7%) and complied with referrals (61.7%) compared to men. Among the referred individuals tested for TB at the referral facility, around 5% were diagnosed with TB. In the qualitative study, we revealed that factors such as competing daily activities at home, physical disability, being non-ambulatory, community stigma, personal negligence, low self-worth associated with addiction, lack of adequate awareness and misconceptions about TB disease, and financial problems were found to be the commonest reasons for non-compliance to referral. On the other hand, we identified severe disease symptoms, HEW’s recommendations, and social pressure as reasons enhancing compliance to referral.

The effectiveness of the presumptive TB case referral partly depends on the appearance of most if not all referred cases to the facility for testing and treatment initiation if they are found to have TB. This study showed that nearly 49% of the referred presumptive TB cases didn’t comply to the referral by the HEWs. This is worrisome as it could contribute to higher missed TB cases and leads to a lower-case notification rate. Low case detection poses a threat of continued transmission of TB in the community [23]. The proportion of non-compliance after referral is higher in this study compared to a study in Vietnam. In a scheme involving presumptive TB case referral by private facilities, it was found that about 30% of the referred cases didn’t attend the facilities under the Vietnamese National TB Program [25]. In another study conducted in China on presumptive case referral, 93.1% of those referred did arrive at the designated facilities and this high compliance could be due to the robustness of the system put in place for active case finding in the country [26].

In this study, only 48% appeared to the referral facility among the referred cases from households having a known TB case. As household members with a TB case in a household are at a higher risk of contracting the disease compared to the general population [3, 13, 16], most of the referred cases should have appeared to the referral facility for testing. This could be due to, the insufficiency of information provided by the HEWs during referral or inadequate attention given for TB contact tracing. Part of the activities conducted by HEWs in community TB control is health education regarding the disease to increase awareness [22].

This study has also indicated that more than two third of the referred presumptive TB cases were women. Two women were referred for every man referred. And 61% of those who complied to referral were women, consistent with the Chinese study [26]. One possible explanation for this in the study area is that women were more likely to be around home compared to men during HEWs regular house to house visit. Another reason could be that generally women were more likely to obey to recommendations by HEWs. Regardless, this finding showed that HEWs referral of presumptive TB cases could help to increase case finding among women. In Ethiopia, case detection rate is usually lower in women compared to men due to various factors including low health seeking behavior [19]. Other studies involving gender differences in TB case finding and treatment initiation also confirm that passive case finding strategies perform poor among women compared to men [5, 27, 28].

Presence of chronic cough and chest pain symptoms and year of referral independently predicted compliance to referral in this study. The decrease in compliance in the year 2020 compared to the earlier years might be linked with the general decline in health service utilization during the earliest months of the Covid-19 pandemic in Ethiopia [29]. Other symptoms such as having a weight loss, loss of appetite, blood in sputum, fever and presence of TB case in a household had no significant association with compliance to referral. However, in our qualitative findings, severe symptoms including persistent cough, observing blood mixed sputum, progressive weight loss and inability to eat and/or drink had been frequently mentioned as reasons for seeking immediate medical attention and compliance to referral. Studies show that referred cases with less severe disease may not feel the urgency to attend a healthcare facility unlike presumptive TB cases with a severe disease in which case prompt action is taken [2, 26].

Fear of stigma form community members, financial problems and lack of transport had all been described as hinderances for compliance to referral as has been described by other studies as reasons for non-appearance or delayed care seeking for TB diagnosis [2, 14, 15, 24, 30]. Lack of awareness about TB was the other common reason described for non-compliance. These included unawareness about the seriousness of the disease and availability of treatment, the transmissibility of the disease to others with frequent contact, not knowing that TB diagnosis and treatment are free of charge. Previous studies conducted on factors causing delays to seeking care and barriers to TB diagnosis uphold our findings [2, 24, 26]. Apart from these, less frequently mentioned reasons for non-compliance were substance use, giving priority for daily activities, low self-worth, concealing health problems and not seeking advice of others close by and repeated travel to facilities for lab testing and result and treatment initiation. Repeated travel could be a reason for loss to follow up before or after diagnosis specially when the distance to be travelled is long and/or is costly. TB referral facilities could eliminate such hurdles for clients through various ways.

Our study found that 5% of the referred cases (10.8% among the compliers) were diagnosed to have TB. This is a huge addition to the case notification of the area. But, the number of cases found could be increased if diagnostic capacities in the referral facility are improved. Currently, only Acid-fast bacillus (AFB) smear microscopy is used to test sputum. It’s well documented that this test has low sensitivity and could lead to missed cases specially those having a paucibacillary disease [16]. Introduction of additional tests such as GeneXpert in to TB referral facilities could significantly improve the yield and would avoid the need for repeated travel.

The main strength of this study is the use of several distinct data sources both in health posts and a referral facility to help identify the status of presumptive TB cases compliance to referral which otherwise could not be identified in a single register or database. This study is also the first of its kind to evaluate a program effectiveness and could act as evidence for decision making and possible implication for policy changes with regards to presumptive TB cases referral. One limitation of this study was the reliance on secondary data from referral registers and unit TB registers which had restricted the number and types of variables included in the quantitative data set. The other limitation was the presence of irregularities and lack of organization in the documentation of referred presumptive TB cases who arrived in the referral facility which might have caused under estimation of compliance to referral.

## Conclusion

Our finding revealed that there was a high level of non-compliance to referral among presumptive TB cases referred by HEWs. Factors such as lack of adequate awareness about TB, severe disease symptoms, facing social issues in the family and/or community, financial and transportation problems affect compliance to referral. Hence, measures such as improving awareness among the community members about the disease including its symptoms and transmissibility, informing availability of free treatment, addressing misconceptions, providing support for the elderly and disabled, and checkup visits by HEWs to the households of presumptive TB cases after referral could improve compliance. Future interventions may aim at bringing some services such as screening and sputum collection closer to the households of the presumptive TB cases to address financial and transportation issues and problems faced by non-ambulatory individuals. The higher referral and compliance of women by the referral scheme is desirable and needs to be strengthened.

## Electronic supplementary material

Below is the link to the electronic supplementary material.

Supplementary Material 1

## Data Availability

The raw dataset could be obtained from the corresponding author on a reasonable request.

## Abbreviations

AOR: Adjusted Odds Ratio
CI: Confidence Interval
COR: Crude Odds Ratio
EPHI: Ethiopian Public Health Institute
EPTB: Extrapulmonary Tuberculosis
HEP: Health Extension Program
HEW: Health Extension Worker
HIV: Human Immuno-deficiency Virus
MDR: Multidrug Resistant
RHB: Regional Health Bureau
TB: Tuberculosis
