# Supplementary material for: High non-compliance rate among presumptive tuberculosis cases referred from peripheral health facilities in silti district of Southern Ethiopia: a mixed methods study
Source: Arch Public Health. 2023 Apr 4;81:50. doi: 10.1186/s13690-023-01071-w (PMC10071467; doi:10.1186/s13690-023-01071-w)
Supplement: Supplementary file 1 — Supplementary Material 1 [file 13690_2023_1071_MOESM1_ESM.docx]

**Aims and Scope statement**

1. What is known?

Of the estimated 10 million people who develop TB annually, about one-third of them are missed during diagnosis. As a result, they are not notified and do not get the recommended chemotherapy early. Most such patients seek care at a delayed time leading to worse treatment outcomes and increased cost of care for the patients and families. Untreated patients, especially those with cavitary pulmonary TB, are also likely to transmit the infection to other household and community members thereby maintaining the transmission chain. Hence, redoubling efforts to improve early identification and treatment are key elements of improved TB care and prevention. On the other hand, relying on TB case detection through self-referral of patients with serious symptoms to health care facilities (also known as Passive Case Finding) has been consistently shown to be ineffective in closing the missed cases gap in the community. Furthermore, it is known to cause gender disparity in case detection, particularly in rural areas where women do not have enough empowerment to seek care for their health. The WHO recommends Active Case Finding (ACF) through systematic screening of selected high-risk groups (such as contacts of TB patients, people living with HIV, people with Diabetes, elderly and malnourished people, and miners exposed to silica) to detect cases early and to promptly initiate treatment. Identification and referral of Presumptive TB cases /TB suspects/ for diagnosis is known to improve case detection and is considered an ACF approach. Primary healthcare workers can play an important role in suspect identification and referral. In Ethiopia, Health Extension Workers are tasked with identifying and referring individuals with symptoms suggestive of TB during their routine work both in Health Posts and while conducting house-to-house visits in the community. Despite its implementation since 2006, evaluation of its effectiveness including the level of compliance/non-compliance to referral and the possible reasons behind it has never been done.

1. What does the study add?

This is the first study on the effectiveness of community-based presumptive TB case referral in Ethiopia, which is one of the 30 high TB burden countries in the world. In this study, we estimated the proportion of compliance to a referral of presumptive cases by identifying those who have presented themselves to the referral facility over a specific period, identified the common reasons causing non-compliance with referral, and evaluated the yield of TB among the referred cases. Through a cross-sectional study, we showed that a high proportion of referred presumptive cases did not present themselves to the referral facility. We also found that more women were referred and complied with referrals compared to men. Among the referred individuals tested for TB at the referral facility, around 5% were diagnosed with TB. Furthermore, through employing key informant interviews, we have found factors such as competing daily activities at home, physical disability, being non-ambulatory, community stigma, personal negligence, low self-worth associated with addiction, lack of adequate awareness and misconceptions about TB disease, and financial problems were found to be the commonest reasons for non-compliance to referral.

1. What are the implications for clinical practice, public health and/or research?

The findings from our research could inform the National TB Program of Ethiopia to rethink the way presumptive TB case referral from the community is being implemented. In its current form, the referral implementation is not effective enough in ensuring the diagnostic testing of most if not all referred cases. Hence, actions should to be taken on the identified factors hindering compliance to referral. This could increase the pool individuals with higher likelihood of having TB presenting themselves to referral facilities. This in turn could help increase early detection and treatment of cases and reduce the likelihood of missed TB cases which are the source of continued transmission in the community.
